# Electrochemical fingerprinting sensor for plant phylogenetic investigation: A case of sclerophyllous oak

**DOI:** 10.3389/fpls.2022.962301

**Published:** 2022-11-09

**Authors:** Jun Hu, Yin Shen, Yuhong Zheng, Wei Zhou, Hassan Karimi-maleh, Qing Liu, Li Fu

**Affiliations:** ^1^ CAS Key Laboratory of Mountain Ecological Restoration and Bioresource Utilization & Ecological Restoration and Biodiversity Conservation Key Laboratory of Sichuan Province, Chengdu Institute of Biology, Chinese Academy of Sciences, Chengdu, China; ^2^ University of Chinese Academy of Sciences, Beijing, China; ^3^ Key Laboratory of Novel Materials for Sensor of Zhejiang Province, College of Materials and Environmental Engineering, Hangzhou Dianzi University, Hangzhou, China; ^4^ Institute of Botany, Jiangsu Province and Chinese Academy of Sciences (Nanjing Botanical Garden, Memorial Sun Yat-Sen), Nanjing, China; ^5^ School of Resources and Environment, University of Electronic Science and Technology of China, Chengdu, China; ^6^ Department of Chemical Engineering and Energy, Laboratory of Nanotechnology, Quchan University of Technology, Quchan, Iran; ^7^ Department of Chemical Sciences, University of Johannesburg, Doornfontein Campus, Johannesburg, South Africa

**Keywords:** electrochemical fingerprint, phytochemistry, electrochemical sensor, pattern recognition, phylogenetics

## Abstract

Electrochemical fingerprinting can collect the electrochemical behavior of electrochemically active molecules in plant tissues, so it is regarded as a new plant analysis technology. Because the signal of electrochemical fingerprinting is positively correlated with the amount and type of electrochemically active molecules in plant tissues, it can also be used to reflect genetic differences between different species. Previous electrochemical fingerprinting techniques have been frequently used in phylogenetic studies of herbaceous plants. In this work, 19 *Quercus* species (17 evergreen or semi evergreen species and 2 deciduous species) were selected for investigation. The results indicated the electrochemical fingerprint of some species share similar features but can be distinguished after changing the recording condition (extraction solvent and electrolyte). The two sets of electrochemical fingerprint data can be used to construct different pattern recognition technology, which further speeds up the recognition efficiency. These electrochemical fingerprints were further used in phylogenetic investigations. The phylogenetic results deduced from electrochemical fingerprinting were divided mainly into three clusters. These can provide evidence for some of these arguments as well as new results.

## Introduction

Electrochemical fingerprinting is a new analytical technique, which can be used to collect the information of electrochemically active molecules in plant tissues (Xu et al., 2020; Zhou et al., 2020; Fan et al., 2021; Fu et al., 2021; Wang et al., 2021; Zheng et al., 2021b; Fu et al., 2022). The fingerprint can be used not only for plant identification and growth monitoring, but also for phylogenetic investigation in recent years. These applications are due to the fact that the type and amount of electrochemically active substances in plant tissues can reflect their differences at the genetic level (Karimi-Maleh et al., 2021b; Karimi-Maleh et al., 2021a). Thus, this new analytical technique is beginning to serve as a complementary methodology for phytochemical studies and plant phylogeny. Today, research on electrochemical fingerprinting is mainly focused on herbaceous and lianas. This is because the active substances in these plant tissues can be easily extracted and can contribute a distinct electrical signal. In contrast, there have been few reports on woody plants based on electrochemical fingerprinting. This is because woody plants have more cellulose and lignin in their tissues, which reduces the accuracy of electrochemical fingerprinting.

*Quercus* is a family of trees under Fagaceae. It is widely distributed in Asia, Africa, Europe and America and other regions, there are about 500 species. *Quercus* are mainly distributed in tropical (subtropical) and temperate regions of the Northern Hemisphere, and are important species of broad-leaved forests. It occupies a large share in the forest area of the northern hemisphere and is of great significance to the local environment beautification and ecological restoration. Abrams believes that *Quercus* has developed root system and thick leaves, which can keep high water potential of these plants in the case of drought and prevent wilting (Abrams, 1990). In addition, *Quercus* can maintain a higher photosynthetic rate under low water potential compared with other groups in the same domain, which is conducive to obtaining competitive advantages in the environment. Cavende-bares et al. (Cavender-Bares et al., 2004b; Cavender-Bares et al., 2004a) found that the life history characteristics of *Quercus* were also influenced and restricted by their living environment. For example, *Quercus* adapted to post-fire habitat are generally shrub type, with strong redifferentiation ability of rhizomes. *Quercus* that grow in humid environment are generally tall trees. *Quercus* distributed in arid environment have resistance mechanism to xylem catheter embolization, which leads to the small amount of water passing through, which is a response of plants to drought stress. *Quercus* grow in humid environments and are free to absorb large amounts of water.

The name *Quercus* was first suggested by the Swedish naturalist Linnaeus. In 1867, Swedish botanist Oersted (Øersted, 1867) distinguished *Cyclobalanopsis* Oerst from other plants in the genus based on the conformation of shell bracts into concentric circles, and became an independent species of *Cyclobalanopsis* Oerst. In 1924 Trelease (Trelease, 1924) divided *Quercus* into six subgenus groups, including *Cyclobalanopsis*, *Cerris*, *Erythrobalanus*, *Protobalanus* and other subgenera. Camus (Chevalier and Camus, 1954) divided *Quercus* into two subgenera, subgen. *Euquercus* and Subgen. *Cyclobalanopsis*. In 1993, Nixon (Nixon, 1993) adjusted some species of *Quercus* and divided *Quercus* into subgenera Subgen. *Quercus* and subgen. *Cyclobalanopsis*. Japanese scholar Shimaji thought that *Quercus* could be divided into three subgenera after analyzing the anatomical characteristics. It includes subgen. *Erythrobalanus*, subgen. *Lepidobalanus* and subgen. *Cyclobalanopsis*, respectively (Shimaji, 1962). Molecular evidence suggests that there are six large groups of present-day *Quercus*, namely group *Lobatae* (Red Oaks), group *Protobalanus* (Intermediate Oaks), group *Ilex*, group *Cerris*, group *Quercus* (White Oaks) and group *Cyclobalanopsis* (now a separate genus) (Manos et al., 1999; Manos and Stanford, 2001; Denk and Grimm, 2010). In China, botanists have divided *Quercus* into five groups according to the phylogeny and quantitative classification (Peng et al., 2007). They are sect. *Engleriana*, sect. *Brachylepids*, sect. *Quercus*, sect. *Echinolepides* and sect. *Aegilops*. Among them, sect. *Quercus* and sect. *Aegilops* usually deciduous broad-leaved plants, others are evergreen or semi-evergreen broad-leaved plants. Most of evergreen species are called sclerophylla oak because their leaves are leathery, hard and spiny (Chaudhri et al., 2022; Farooq et al., 2022; Ismail et al., 2022). There has been a great controversy about the relationship between species within the sclerophylla oak. In this work, 19 species of Quercus (17 evergreen or semi evergreen species and 2 deciduous species) and 2 species of Cyclobalanopsis were investigated using electrochemical fingerprinting. Electrochemical fingerprinting of all plant tissues was collected under two conditions. The collected results were used not only for plant identification, but also for phylogenetic investigation.

## Materials and method

### Sample collection

Leaves of *Quercus rehderiana*, *Q. monimotricha, Q. gilliana, Q. variabilis, Q. pseudosemecarpofolia, Q. guajavifolia, Q. longispica, Q. spinosa, Q. engleriana, Q. fimbriata, Q. franchetii, Q. senescens, Q. baronii, Q. dolicholepis, Q. cocciferoides, Q. oxyphylla* and *Q. aquifolioides* were collected during field investigation*, Q. aliena, Q. phillyraeoides, Cyclobalanopsos myrsinifolia* and *Cyclobalanopsis glauca* were collected from Chengdu City Park and Nanjing Botanical Garden Men. Sun Yat-Sen (**Table 1**). The voucher specimens are deposited in the herbarium of Chengdu Institute of biology (CDBI), Chinese Academy of Sciences and the herbarium of Nanjing Botanical Garden Men.Sun Yat-Sen (NAS). Only mature and healthy leaves were harvested. All samples were kept frozen (-20°C) before analysis.

**Table 1:** Leaves for electrochemical analysis.

NO	species	Collector	Voucher	Location
1	Q. monimotricha	Jun Hu, et all	CDhujun20210713P01S01	Meigu, Sichuan
2	Q. guajavifolia	Jun Hu, et all	CDhujun20210714P02S02	Meigu, Sichuan
3	Q. aquifolioides	Jun Hu, et all	CDhujun20210804P02S01	Meigu, Sichuan
4	Q. senescens	Jun Hu, et all	CDhujun20210717P02S01	Xichang, Sichuan
5	Q. rehderiana	Jun Hu, et all	CDhujun20210724P01S01	Yongsheng, Yunnan
6	Q. longispica	Jun Hu, et all	CDhujun20210721P01S01	Huaping, Yunnan
7	Q. pseudosemecarpifolia	Jun Hu, et all	hujun20210718-B02	Ninglang, Yunnan
8	Q. cocciferoides	Jun Hu, et all	CDhujun20210727P02S01	Muli, Sichuan
9	Q. fimbriata	Jun Hu, et all	CDhujun20210808P02S01	Kangding, Sichuan
10	Q. variabilis	Jun Hu, et all	CDhujun20210809P01S08	Mianning, Sichuan
11	Q. spinosa	Jun Hu, et all	CDluoyao20210914S002	Lixian, Sichuan
12	Q. dolicholepis	Jun Hu, et all	CDluoyao20210914S003	Lixian, Sichuan
13	Q. baronii	Jun Hu, et all	CDluoyao20210914S001	Lixian, Sichuan
14	Q. engleriana	Sirong Yi	yisirong20210911B01	Zunyi, Guizhou
15	Q. oxyphylla	Sirong Yi	yisirong20210905B01	Shizhu, Chongqing
16	Q. franchetii	Jun Hu, et all	hujunCX001	Huili, Sichuan
17	Q. aliena	Yao Luo	None	Chengdu city park, cultivated plants
18	Q. gilliana	Jun Hu, et all	CDhujun20210809P01S01	Mianning, Sichuan
19	Q. phillyraeoides	Yuhong Zheng	NAS00590201	Nanjing Botanical Garden Men.Sun Yat-Sen, cultivated plants
20	Cyclobalanopsis glauca	Yuhong Zheng	None	Nanjing Botanical Garden Men.Sun Yat-Sen, cultivated plants
21	C. myrsinifolia	Yuhong Zheng	None	Nanjing Botanical Garden Men.Sun Yat-Sen, cultivated plants

### Extraction preparation

All extraction process was conducted under room temperature. Water and ethanol were used as the solvents in the extraction procedure. Specifically, 0.3 g leaves were cut and added to 5 mL of solvent. The mixture was supplemented with four grinding beads. The tube was put in a tissue grinding device (Meibi-96, Zhejiang, China) for 2 min extraction. After waiting for precipitation, the supernatant was collected for electrochemical fingerprint collection.

### Electrochemical fingerprints collection

Phosphate buffer solution (PBS, 0.1 M) and acetic acid buffer (ABS, 0.1 M) were used as electrolytes to support electrochemical fingerprint collection. Electrochemical fingerprinting was determined using a traditional three-electrode system. A glassy carbon electrode, a platinum wire and an Ag/AgCl electrode were used as working electrode, counter electrode and reference electrode, respectively. All electrochemical experiments were conducted under a CHI 760E working station at room temperature. a differential pulse voltammetry (DPV) was recorded from -0.1 to 1.3 V. The experimental data was then normalized for further analysis.

## Results and discussion

**Figure 1:**
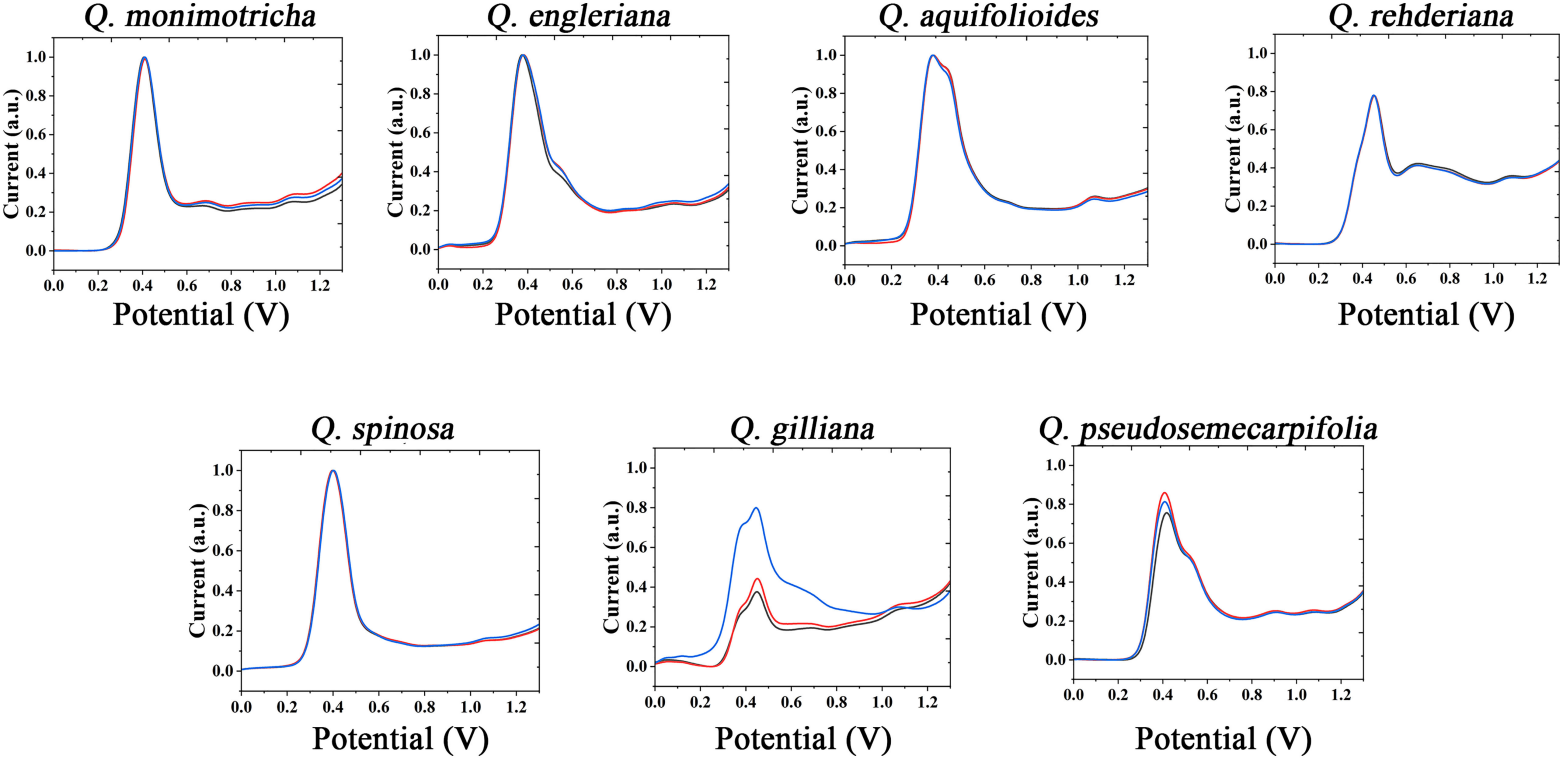
Electrochemical fingerprint of *Q. monimotricha, Q. engleriana, Q. aquifolioides, Q. rehderiana, Q. spinosa, Q. gilliana* and *Q. pseudosemecarpifolia* after water extraction and recorded under PBS condition with three repetitive tests.

The information of electrochemically active molecules in plant tissues was collected by electrochemical fingerprinting. The anodic scanning representing the electrochemical oxidation behavior of molecules. As shown in **Figure 1** (collected under PBS after extraction with water), in the range of 0-1.3V, *Q. monimotricha, Q. engleriana, Q. aquifolioides, Q. rehderiana, Q. spinosa, Q. gilliana* and *Q. pseudosemecarpifolia* all showed electrochemical oxidation behavior. In addition, these species show similar electrochemical oxidation trend, with an obvious electrochemical oxidation peak near 0.4 V. This similarity in electrochemical behavior is common among plants of the same genus. This is because species within the genus have relatively similar genes, and therefore have a high degree of similarity in the species of electrochemically active molecules in tissues (Ye et al., 2021). However, beyond the obvious large oxidation peak of about 0.4 V, different species showed different electrochemical behaviors. For example, *Q. aquifolioides* has a half-overlapped oxidation peak near 0.4 V. *Q. rehderiana* has a wide oxidation peak at about 0.6 V, representing a range of substances oxidized in this window. On the other hand, there are some species that are very similar in their electrochemical behavior, such as *Q. engleriana and Q. pseudosemecarpifolia*.

**Figures S1** and **S2** show the electrochemical behavior of the remaining species collected under PBS after water extraction. All species exhibit essentially the electrochemical behavior described above. Some of these species exhibit similar electrochemical behavior, such as *Q. oxyphylla* and *Q. variabilis*. Three electrochemical fingerprints were taken for each sample. It can be seen that most of the samples have very good repeatability. However, the DPV curves of some samples do not coincide completely. However, the different fingerprints have a very consistent behavior, representing the same molecules involved in electrochemical oxidation (Zheng et al., 2021a). Some fingerprints had a higher current intensity than others, indicating a higher concentration of one type of electrochemically active molecule in the sample. This is very common in plant samples. Even among plants in the same area, different environmental factors will lead to changes in the content of molecules in tissues (Łaska et al., 2019; Ousaaid et al., 2021).

**Figure 2:**
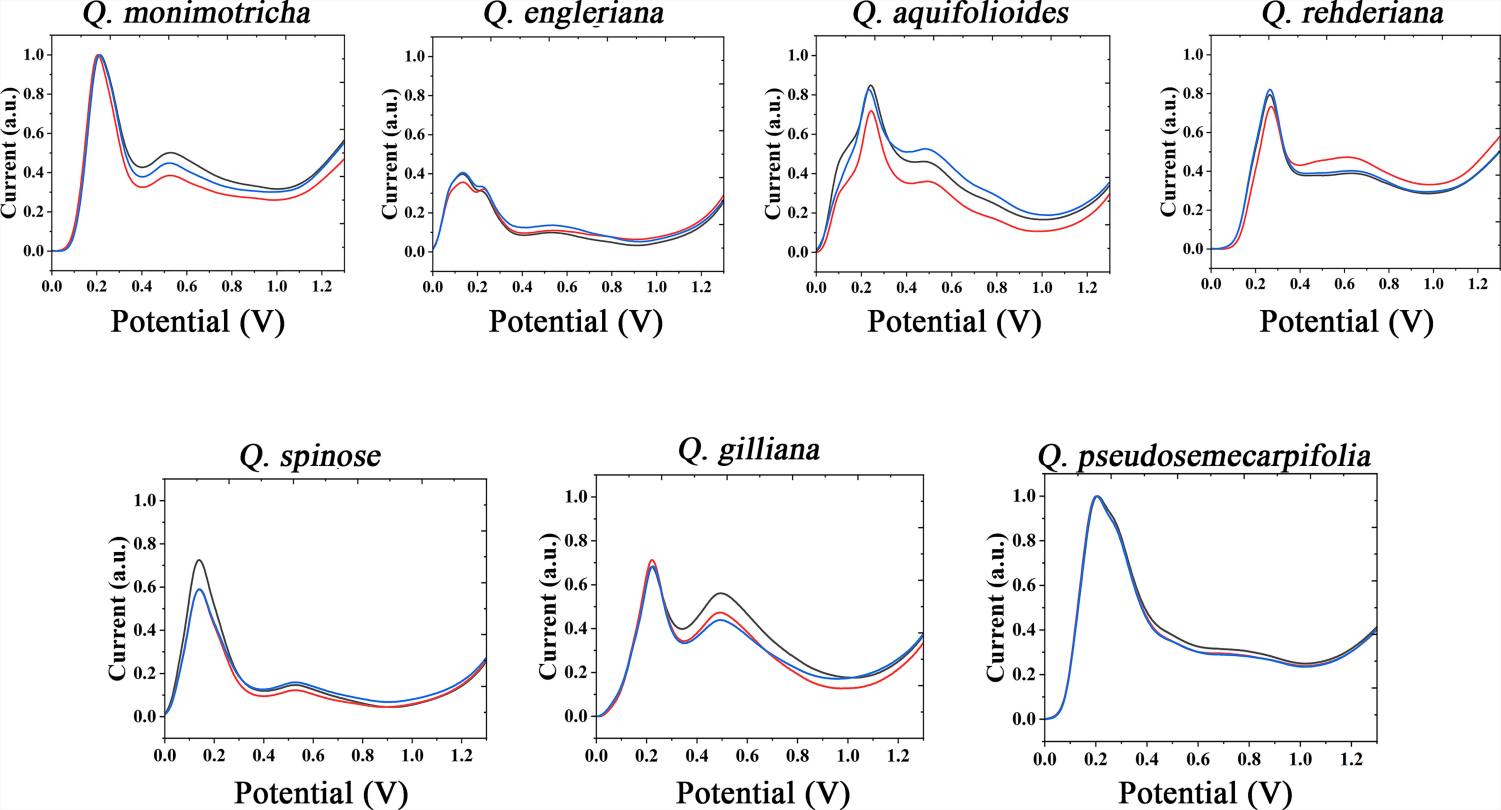
Electrochemical fingerprint of *Q. monimotricha, Q. engleriana, Q. aquifolioides, Q. rehderiana, Q. spinosa, Q. gilliana* and *Q. pseudosemecarpifolia* after ethanol extraction and recorded under ABS condition with three repetitive tests.

The use of the same solvent for plant tissue extraction and the use of the same electrolyte to support electrochemical fingerprinting can cause different species to exhibit relatively similar electrochemical behavior. Therefore, we also extracted the plant tissues with ethanol and collected the electrochemical fingerprints in ABS environment. **Figure 2** shows DPV profiles of *Q. monimotricha, Q. engleriana, Q. aquifolioides, Q. rehderiana, Q. spinosa*, *Q. gilliana and Q. pseudosemecarpifolia* after ethanol extraction under ABS. It can be seen that all species exhibit very different electrochemical behavior under these conditions than in **Figure 1**. There are two factors that account for the difference in electrochemical behavior. First, different solvents extract different electrochemically active molecules from plant tissues (Moro et al., 2020). Therefore, there are different kinds of molecules involved in the process of electrochemical fingerprinting. On the other hand, ABS has an acidic pH, so the same electrochemically active molecule behaves differently in neutral and acidic environments (Fu et al., 2020). As can be seen from the figure, the electrochemical fingerprints similar in **Figure 1**, such as *Q. engleriana* and *Q. pseudosemecarpifolia*, show very large differences in **Figure 2**. Therefore, if the fingerprint of both conditions is combined, the species can be clearly identified. We further conducted MANOVA tests for our data. The p values of the DPVs recorded within species all larger than 0.05, indicating no significant differences. However, when comparing different species, the p-value is between 3.2e^-07^ to 6.9e^-07^. This result suggesting the significantly different. Therefore, the differences of electrochemical fingerprints between species are much larger than the same species. We believe the environmental factors such as habitat and fertilization certainly affect the content of many chemical constitutions (including electro-active compounds) and consequently change signal intensity. Many GC-MS based works also report the content variation of extracted compounds (Jin, 2009; Berkov et al., 2012). These works suggest the environmental factors can only slightly affect the ratio of compounds without changing compound types. Therefore, main pattern of voltammograms collected from different species will be largely unaffected.

**Figures S2** and **S3** show the electrochemical behavior of the remaining species collected under ABS after ethanol extraction. The trends of these electrochemical fingerprints are very similar to those in **Figures S1** and **S2**. Different species showed electrochemical oxidation behavior in the scanning window, indicating that chemically active molecules were involved in the reaction. Different species exhibit different behaviors, representing differences in the composition and amount of electrochemically active molecules in their tissues. These differences can be used not only for rapid identification of species, but also to reflect genetic differences.

**Figure 3:**
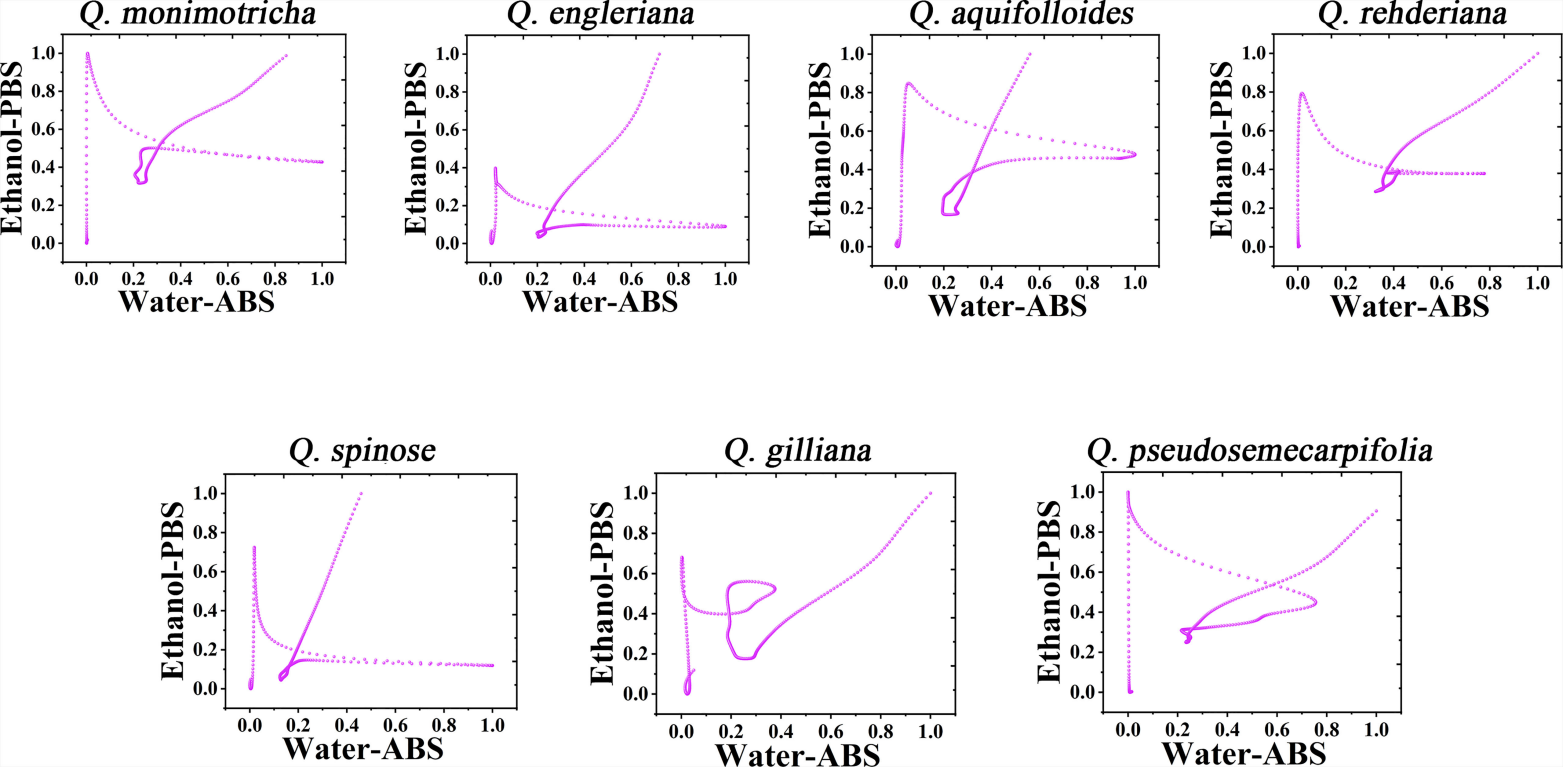
Scatter plots of *Q. monimotricha, Q. engleriana, Q. aquifolioides, Q. rehderiana, Q. spinosa, Q. gilliana* and *Q. pseudosemecarpifolia* combining the signals collected under ABS for the water extracts and under PBS for the ethanol extracts.

It is not a fast method to use DPV curve to identify species, especially when some species have similar profiles. Therefore, it is a scientific way to construct the pattern using electrochemical fingerprint. **Figure 3** shows the scatter plots of *Q. monimotricha, Q. engleriana, Q. aquifolioides, Q. rehderiana, Q. spinosa*, *Q. gilliana and Q. pseudosemecarpifolia* constructed by electrochemical fingerprints collected under two conditions. The data on the X-axis is the current value of the electrochemical fingerprint collected under PBS after water extraction of different species tissues, while the data on the Y-axis is the current value collected under ABS after ethanol extraction of these species. As you can see, when the two sets of fingerprint data are combined, different species present different scatter plots. Scatter plots of the remaining species are provided in **Figures S5** and **S6**. It can be seen that the difference of scatter plots of different species is greater than the direct DPV profile. This is because the superposition of multidimensional data increases the abundance of data and thus improves the resolution (Dexter et al., 2018; Nonato and Aupetit, 2018). Species identification can be achieved by dividing the scatter map and counting the number of points in the region.

**Figure 4:**
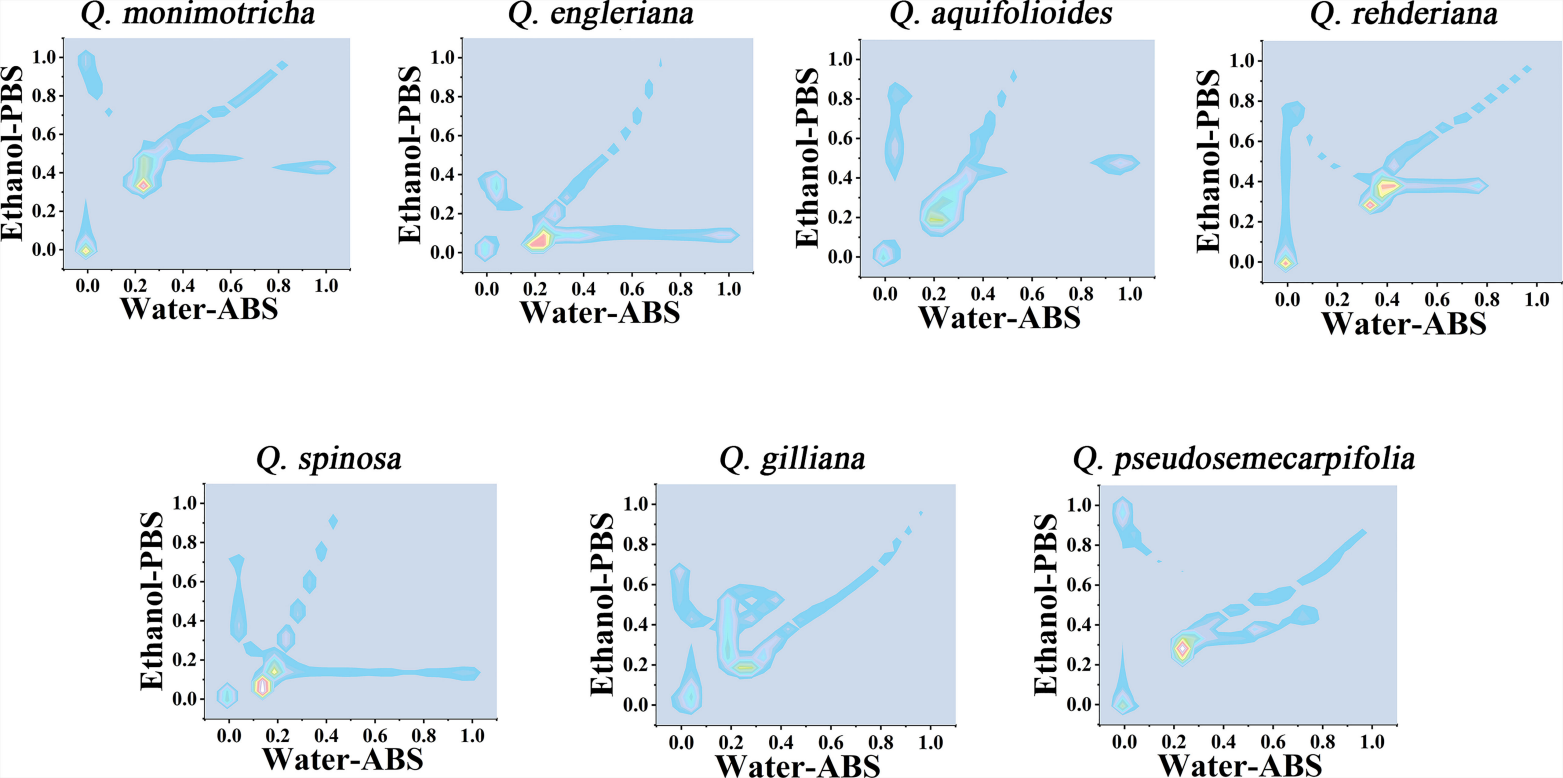
Two-dimensional density map of *Q. monimotricha, Q. engleriana, Q. aquifolioides, Q. rehderiana, Q. spinosa, Q. gilliana* and *Q. pseudosemecarpifolia* combining the signals collected under ABS for the water extracts and under PBS for the ethanol extracts.

Further consideration of the distance between points in the scatter plots can obtain a two-dimensional density diagram. In this mode, areas with multiple points appear bright, while areas with fewer points appear dark. **Figure 4** shows the 3D density plots of *Q. monimotricha, Q. engieriana, Q. aquifolioides, Q. rehderiana, Q. spinosa*, *Q. gilliana and Q. pseudosemecarpifolia* constructed by electrochemical fingerprints collected under two conditions. In this model, differences between species can be identified by targeting the highlighted area directly. For example, the similar *Q. engieriana* and *Q. pseudosemecarpifolia* in **Figure 1** are not the same in the highlighted area here. More specifically, *Q. engleriana* was highlighted in (0.20, 0.03) while *Q. pseudosemecarpifolia* in (0.22, 0.31). 2D density plots of the remaining species are provided in **Figures S7** and **S8**. The similar profiles of *Q. engleriana* and *Q. pseudosemecarpifolia* in **Figure S1** are not the same in the highlighted area here as well. *Q. oxyphylla* was highlighted in (0.38, 0.21) while *Q. variabilis* in (0.25, 0.18).

**Figure 5:**
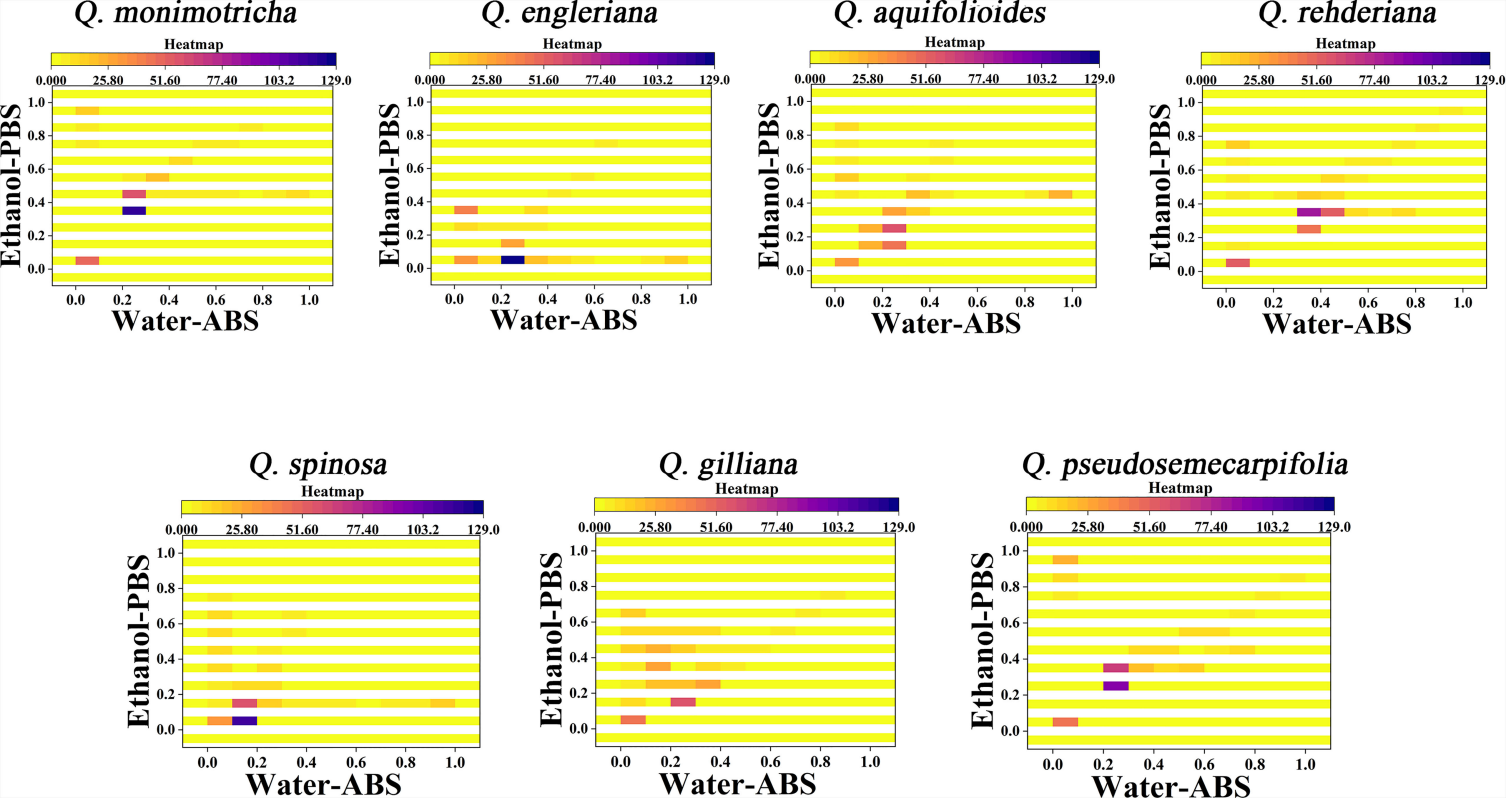
Heatmap of *Q. monimotricha, Q. engieriana, Q. aquifolioides, Q. rehderiana, Q. spinosa, Q. gilliana* and *Q. pseudosemecarpifolia* combining the signals collected under ABS for the water extracts and under PBS for the ethanol extracts.

From the 2D density plots, it was found that the highlights of most species were around 0.2 on the X-axis and 0.4 on the Y-axis. Considering the error of electrochemical fingerprint, this pattern recognition method has a certain probability of misjudgment in theory. However, it was not particularly convenient to determine the unhighlighted areas of the 2D density plots, so we further constructed the heat map using electrochemical fingerprinting. **Figure 5** shows the heatmap of *Q. monimotricha, Q. engieriana, Q. aquifolioides, Q. rehderiana, Q. spinosa*, *Q. gilliana and Q. pseudosemecarpifolia* constructed by electrochemical fingerprints collected under two conditions. Heat maps can be graded according to the density of data points. Moreover, it divides the whole region equally, which is good for statistical purposes (Hervella et al., 2020). According to the scoring method of different grades, heat maps are more suitable for the identification of different species.

**Figure 6:**
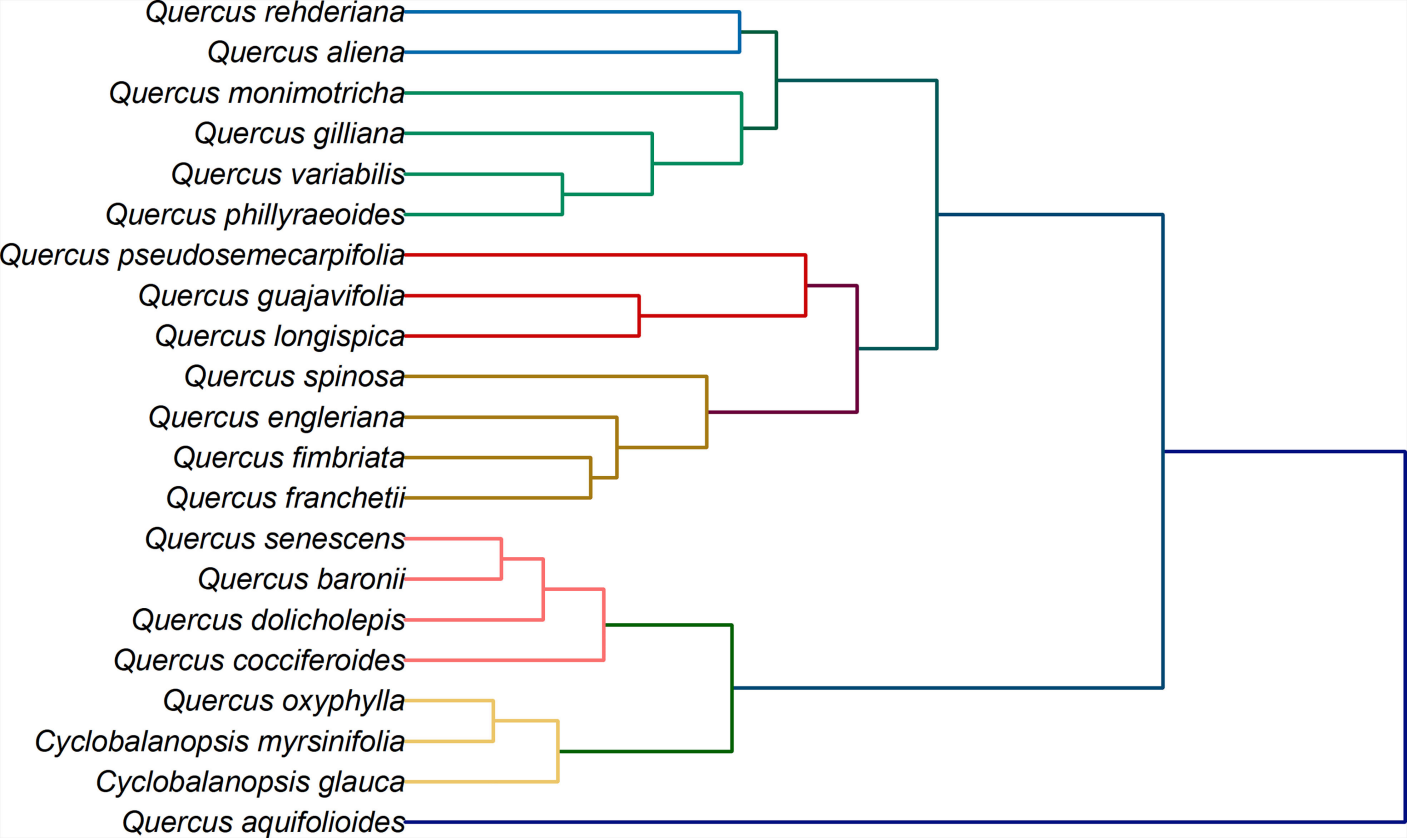
Dendrogram of *Q. rehderiana, Q. aliena, Q. monimotricha, Q. gilliana, Q. variabilis, Q. phillyraroides, Q. pseudosemecarpofolia, Q. guajavifolia, Q. longispica, Q. spinosa, Q. engleriana, Q. fimbriata, Q. franchetii, Q. senescens, Q. baronii, Q. dolicholepis, Q. cocciferoides, Q. oxyphylla, Q. aquifolioides, Cyclobalanopsos myrsinifolia* and *Cyclobalanopsis glauca* based on electrochemical fingerprints.

In addition to species identification, another potential application of electrochemical fingerprinting is to use fingerprint differences to investigate phylogenetic position of species. This is because although electrochemical fingerprinting cannot be used for qualitative and quantitative analysis of active molecules in plant tissues, its current value is proportional to their amount. At the same time, the amount and types of electrochemically active substances in plant tissues are regulated by genes. Therefore, differences in electrochemical fingerprints can be used to reflect genetic differences between different species (Fu et al., 2019; Lu et al., 2020; Sun et al., 2021). The electrochemical fingerprint data under different conditions can be used for cluster analysis after the same processing. The collection of electrochemical fingerprinting for more than one condition can increase the abundance of data and provide a more complete picture of electrochemically active molecules in plant tissues. **Figure 6** shows the clustering analysis graph constructed by using the electrochemical fingerprints collected under the two conditions. It can be seen from the figure that the whole system tree can be divided into five clusters. *Cyclobalanopsis myrsinifolia* and *Cyclobalanopsis glauca* were grouped together as outgroups. This is because they belong to a different genus than other species. However, *Q. oxyphylla* was also grouped together. We did not find previous reports on *Q. oxyphylla* in related phylogenetic studies, so we could not compare this result with previous investigations. This result deduced from electrochemical fingerprinting potentially provides a report for future research. Curiously, *Q. aquifolioides* did not cluster with any other species. In recent years, several molecular markers have been used to study the evolutionary history and population dynamics of *Q. aquifolioides*, and to reveal the influence of past climate and geological history on the intraspectic radiation evolution of this species (Du et al., 2017). Based on chloroplast fragments (cpDNA), ITS sequences, and microsatellite markers (nSSR), Feng et al. (2016) investigated the evolutionary history and population dynamics of *Quercus pseudosemecarpifolia*, *Quercus aquifolioides*, and *Quercus rehderiana* distributed in the Eastern Himalaya-Hengduan Mountains and their adjacent areas. They reveal the effects of the uplift of the Tibetan Plateau and the quaternary ice age upheaval on the distribution pattern of genetic variation of quercus sclerotiorum species in the region. However, due to the limited number of selected molecular markers and the limitations of selected sample materials, the current studies cannot fully explain their genetic evolution. Unfortunately, the results of electrochemical fingerprinting do not provide further evidence. The remaining species are divided mainly into three clusters. The first cluster is the largest cluster, which contains *Q. rehderiana, Q. aliena, Q. monimotricha, Q. gilliana, Q. variabilis* and *Q. phillyraeoides*. Previously, *Q. rehderiana*, *Q. pseudosemecarpifolia* and *Q. gilliana* were merged into a species based on AFLP markers (Zhou et al., 2003). Our results here support the merge of *Q. rehderiana* and *Q. gilliana*, but *Q. pseudosemecarpifolia* is in another cluster. The second cluster includes *Q. pseudosemecarpifolia, Q. guajavifolia, Q. longispica, Q. spinosa, Q. engleriana, Q. fimbriata* and *Q. franchetii*. Ju et al. (2019) reported in recent work that *Q. pseudosemecarpifolia* and *Q. longispica* can be clustered together. They also reported that *Q. guajavifolia* and *Q. spinosa* could be clustered together. The two species *Q. engleriana* and *Q. franchetii* are rarely studied and no taxonomic results have been reported. The third cluster includes *Q. senescens*, *Q. baronii*, *Q. dolicholepis* and *Q. cocciferoides*. This result is partly confirmed by the AFLP analysis (Shuxia et al., 2003; Shuxia et al., 2005). Although many taxonomists have studied *Quercus*, it is difficult to grasp the taxonomic characters of *Quercus* because of its large number of species and wide distribution. Many of these species have great morphological variation in different habitats. At the same time, the phenomenon of interspecific hybridization is common in natural environment, which brings some difficulties to phylogenetic study. Our phylogenetic results here, based on electrochemical fingerprinting, provide evidence for some of these arguments as well as new results. This provides a direction for future phylogenetic studies of *Quercus*.

## Conclusion

The electrochemical fingerprints of *Q. rehderiana, Q. aliena, Q. monimotricha, Q. gilliana, Q. variabilis, Q. phillyraroides, Q. pseudosemecarpofolia, Q. guajavifolia, Q. longispica, Q. spinosa, Q. engleriana, Q. fimbriata, Q. franchetii, Q. senescens, Q. baronii, Q. dolicholepis, Q. cocciferoides, Q. oxyphylla, Q. aquifolioides, Cyclobalanopsos myrsinifolia* and *Cyclobalanopsis glauca* were recorded by their leaf extract under electrolytes. Water and ethanol were selected as extraction solvents while the PBS and ABS were used as supporting electrolyte. The electrochemical fingerprints showed that all *Quercus* showed similar electrochemical oxidation trends, but each species still had its own unique characteristics. Species identification can be achieved by analyzing electrochemical fingerprints. The electrochemical fingerprints collected under the two conditions can be used to form different pattern recognition, which is faster and more effective than DPV profile. Electrochemical fingerprinting is used to construct phylogenetic trees because it can reflect the differences at the genetic level of different species. Our phylogenetic results here, based on electrochemical fingerprinting, provide evidence for some of these arguments as well as new results. This provides a direction for future phylogenetic studies of *Quercus*.

## Data availability statement

The original contributions presented in the study are included in the article/**Supplementary Material**. Further inquiries can be directed to the corresponding authors.

## Author contributions

LF, QL, and HK contributed conception and design of the study. JH, YS, YZ, and WZ conducted electrochemical experiments. JH, YS, and HK performed the statistical analysis. JH, YZ and QL wrote the manuscript. All authors contributed to the article and approved the submitted version.

## Funding

This research was funded by the Second Tibetan Plateau Scientific Expedition and Research Program (STEP) (Grant no. 2019QZKK0301) and Major Program for Basic Research Project of Yunnan Province (202101BC070002).

## Conflict of interest

The authors declare that the research was conducted in the absence of any commercial or financial relationships that could be construed as a potential conflict of interest.

## Publisher’s note

All claims expressed in this article are solely those of the authors and do not necessarily represent those of their affiliated organizations, or those of the publisher, the editors and the reviewers. Any product that may be evaluated in this article, or claim that may be made by its manufacturer, is not guaranteed or endorsed by the publisher.
