# Supplementary material for: Electrochemical fingerprinting sensor for plant phylogenetic investigation: A case of sclerophyllous oak
Source: Front Plant Sci. 2022 Nov 9;13:962301. doi: 10.3389/fpls.2022.962301 (PMC9682139; doi:10.3389/fpls.2022.962301)
Supplement: Supplementary file 1 [file DataSheet_1.docx]

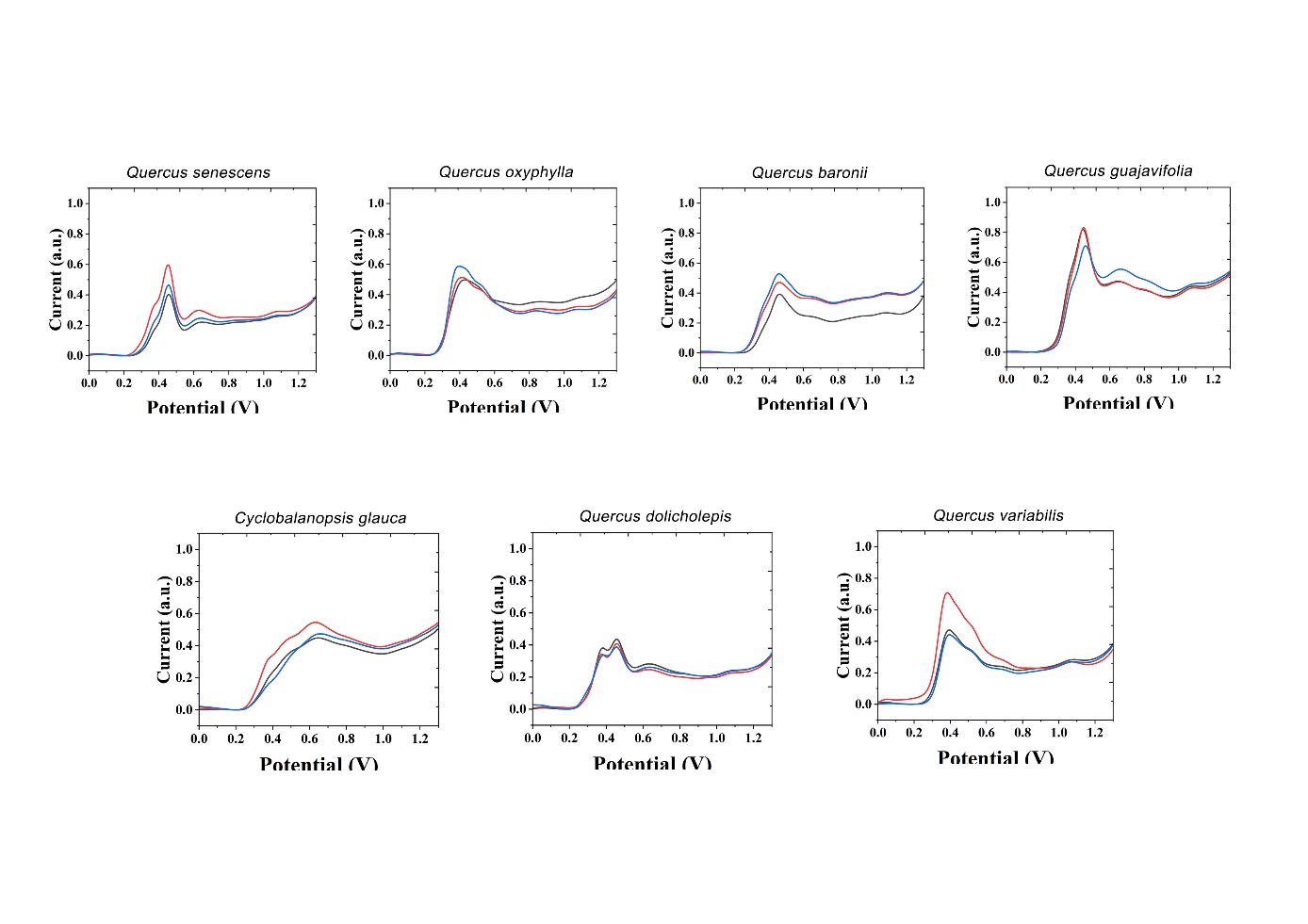


**Figure S1.** Electrochemical fingerprint of *Q. senescens, Q. oxyphylla, Q. baronii, Q. guajavifolia, Cyclobalanopsis glauca, Q. dolicholepis* and *Q. variabilis* after water extraction and recorded under PBS condition.


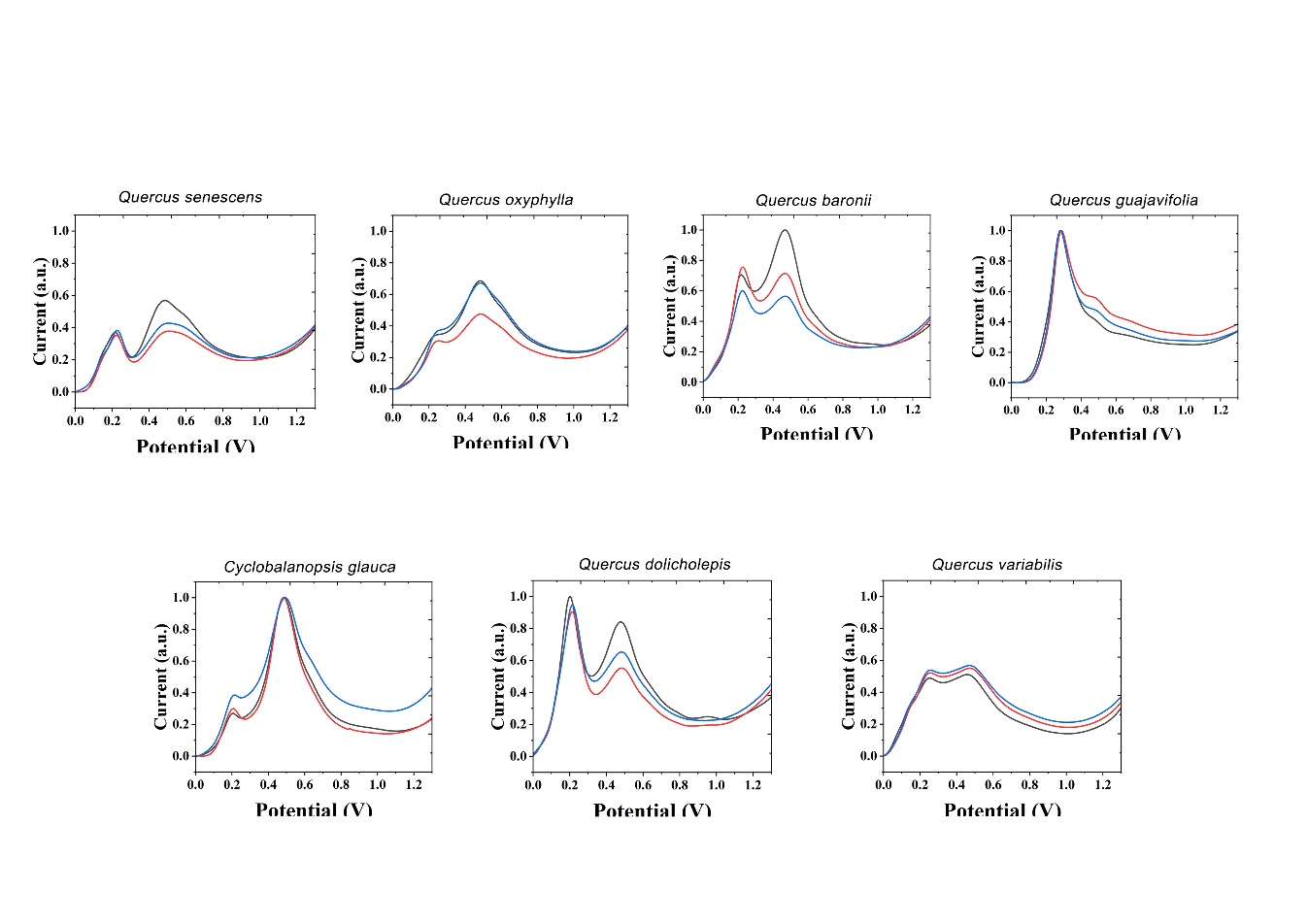


**Figure S2.** Electrochemical fingerprint of *Q. senescens, Q. oxyphylla, Q. baronii, Q. guajavifolia, Cyclobalanopsis glauca, Q. dolicholepis* and *Q. variabilis* after ethanol extraction and recorded under ABS condition.


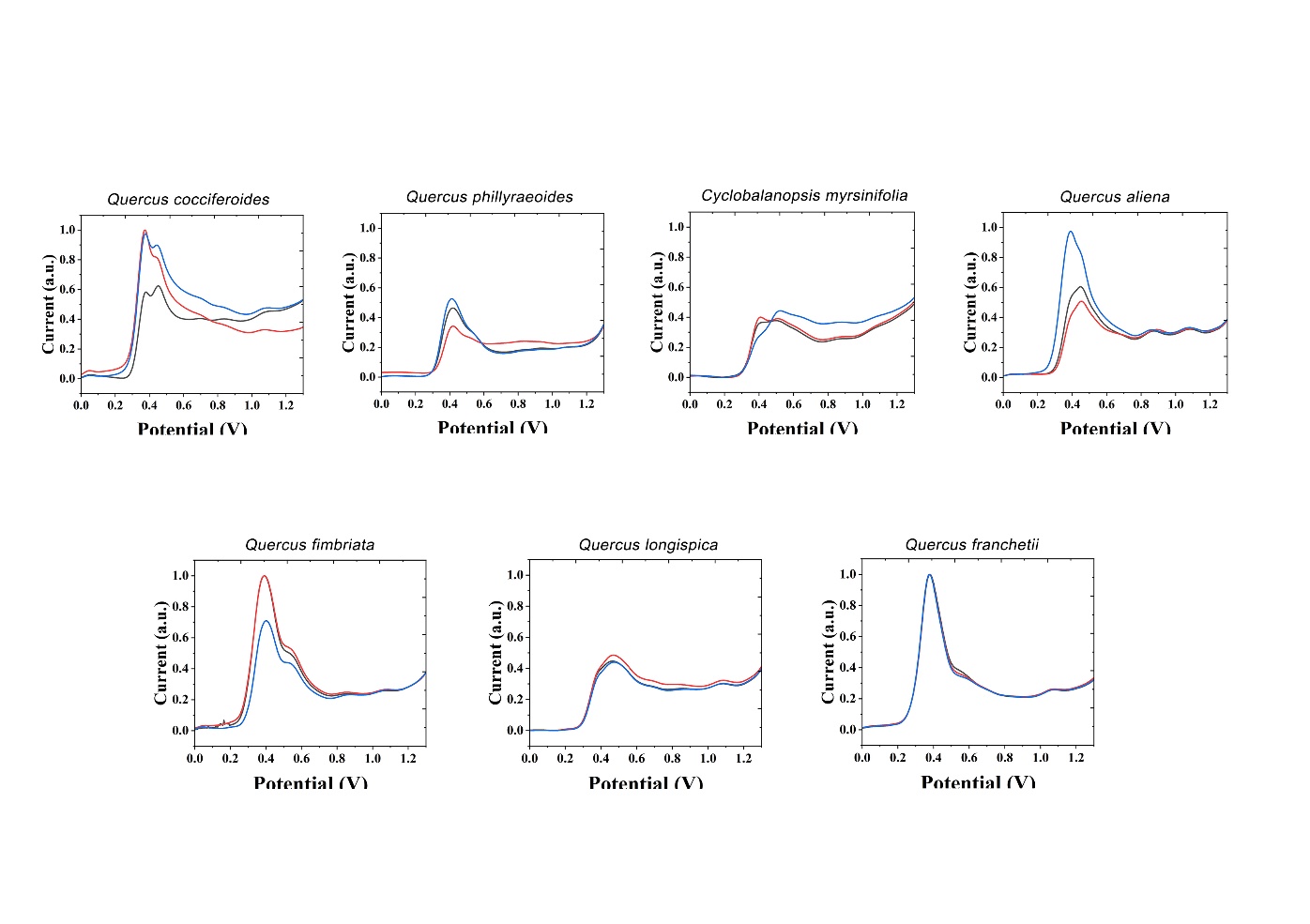


**Figure S3.** Electrochemical fingerprint of *Q.cocciferodies, Q. phillyraeoides, Cyclobalanopsis myrsinifolia, Q. aliena, Q. fimbriata, Q. longispica, Q. franchetli* after water extraction and recorded under PBS condition.


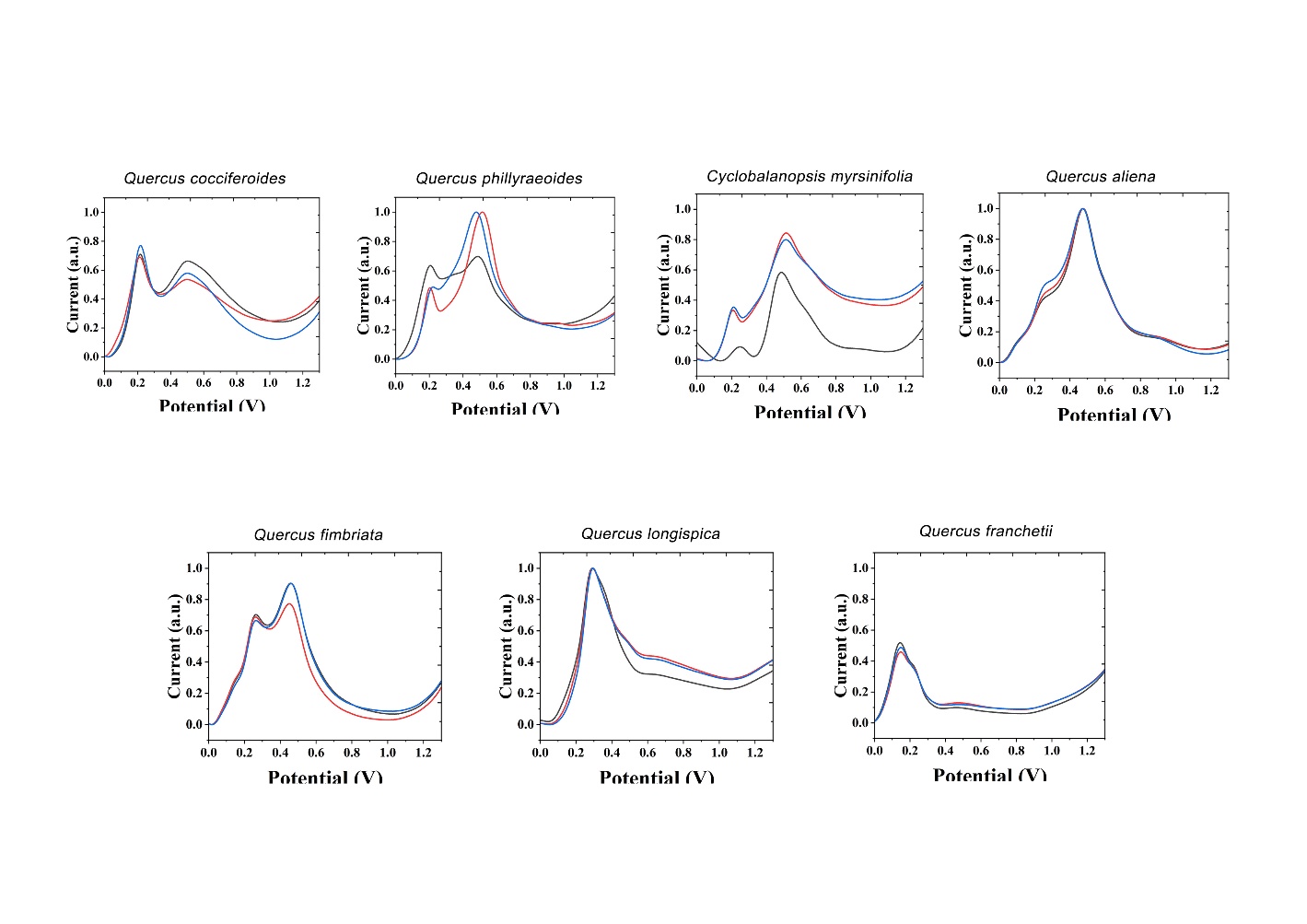


**Figure S4.** Electrochemical fingerprint of *Q.cocciferodies, Q. phillyraeoides, Cyclobalanopsis myrsinifolia, Q. aliena, Q. fimbriata, Q. longispica, Q. franchetli* after ethanol extraction and recorded under ABS condition.


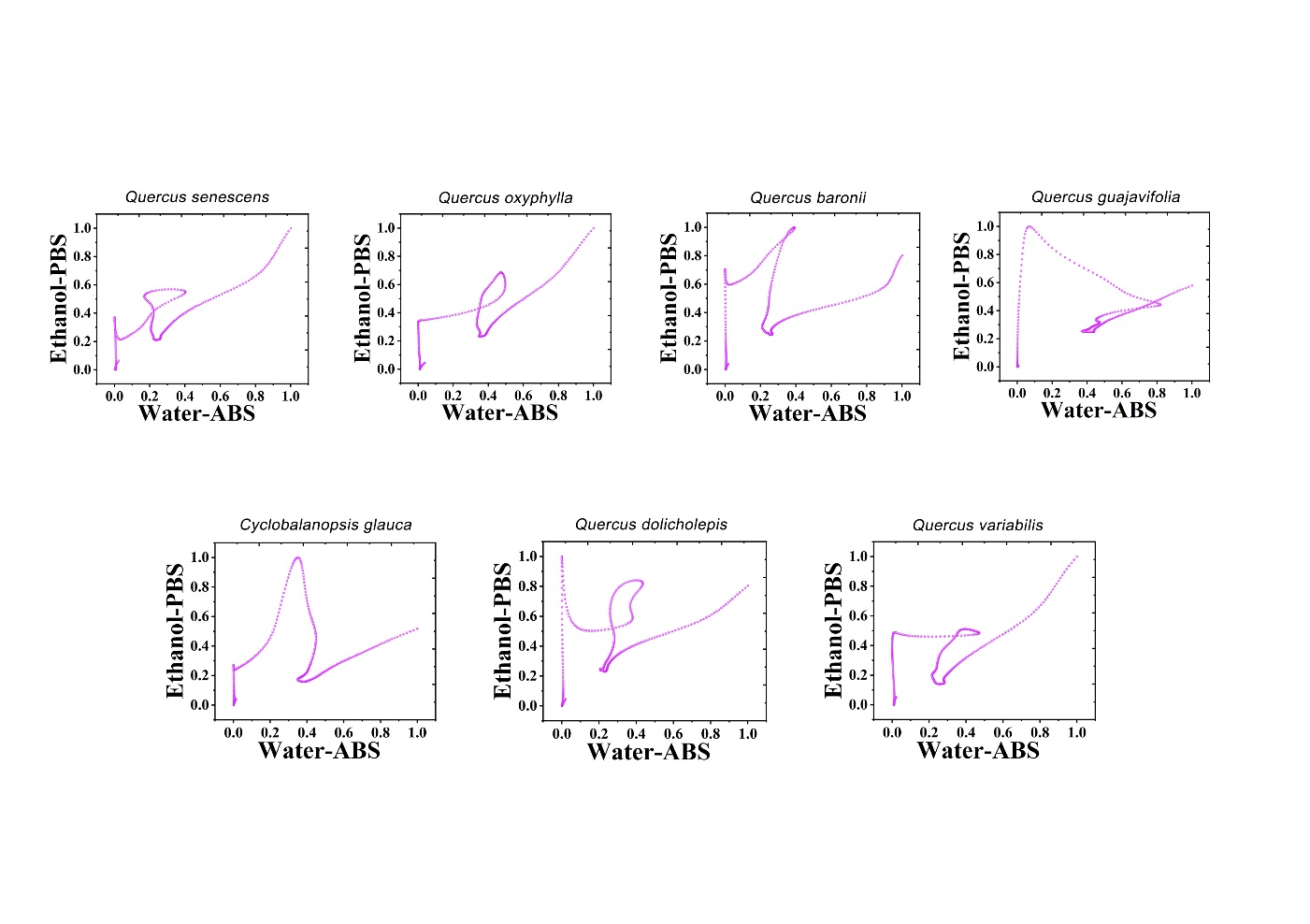


**Figure S5.** Scatter plots of *Q. senescens, Q. oxyphylla, Q. baronii, Q. guajavifolia, Cyclobalanopsis glauca, Q. dolicholepis* and *Q. variabilis* combining the signals collected under ABS for the water extracts and under PBS for the ethanol extracts.


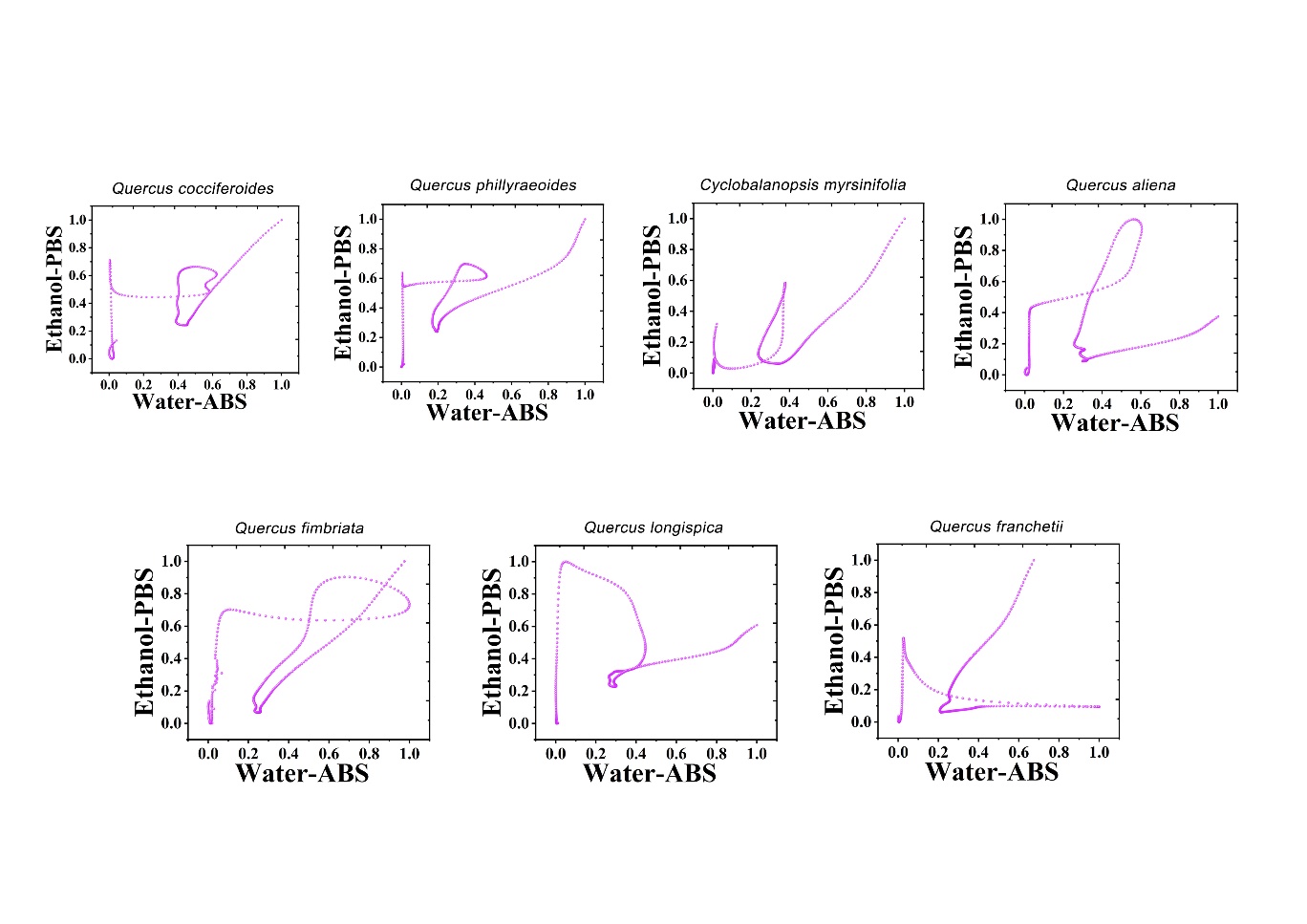


**Figure S6.** Scatter plots of *Q.cocciferodies, Q. phillyraeoides, Cyclobalanopsis myrsinifolia, Q. aliena, Q. fimbriata, Q. longispica, Q. franchetli* combining the signals collected under ABS for the water extracts and under PBS for the ethanol extracts.


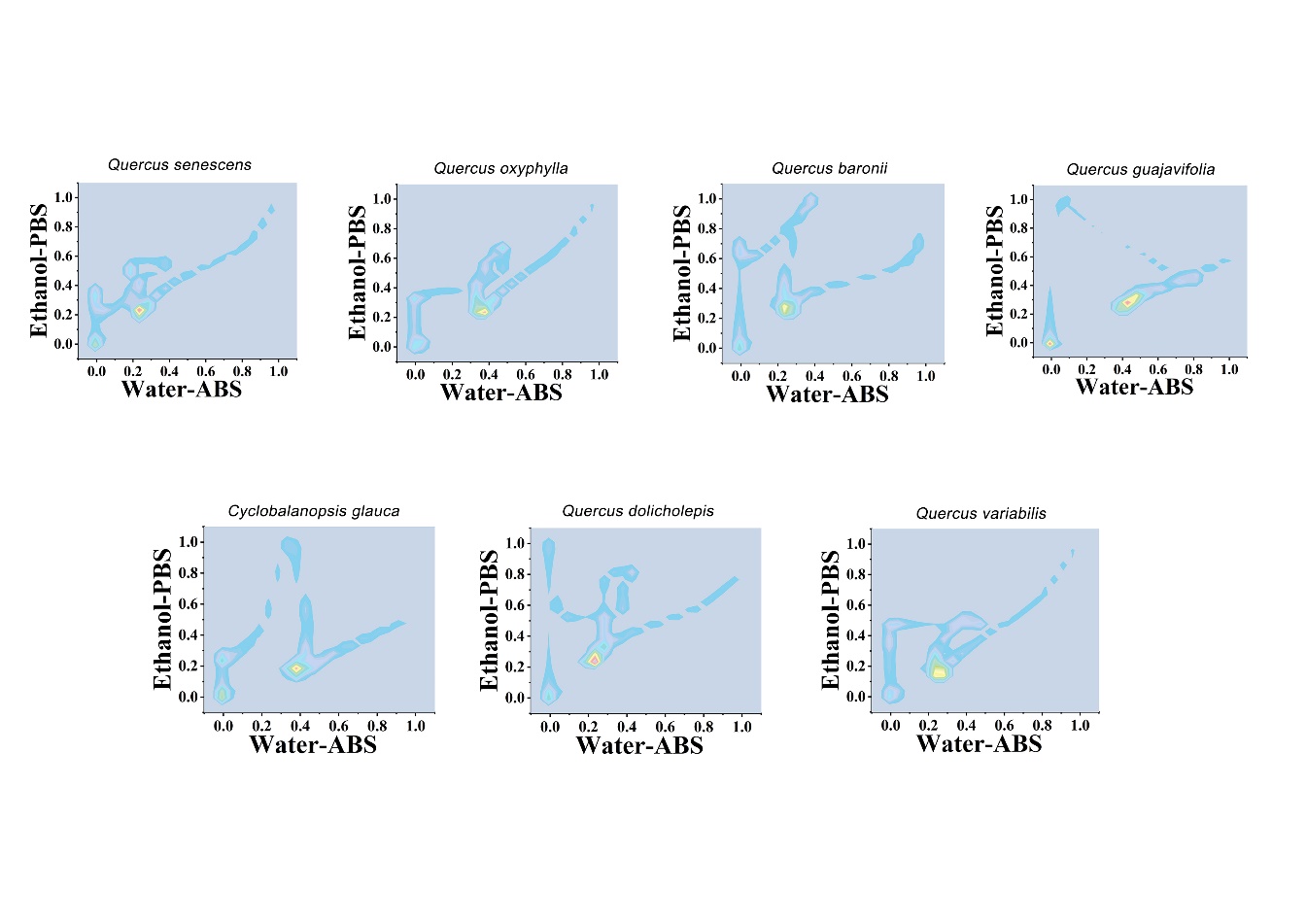


**Figure S7.** Two-dimensional density map of *Q. senescens, Q. oxyphylla, Q. baronii, Q. guajavifolia, Cyclobalanopsis glauca, Q. dolicholepis* and *Q. variabilis* combining the signals collected under ABS for the water extracts and under PBS for the ethanol extracts.


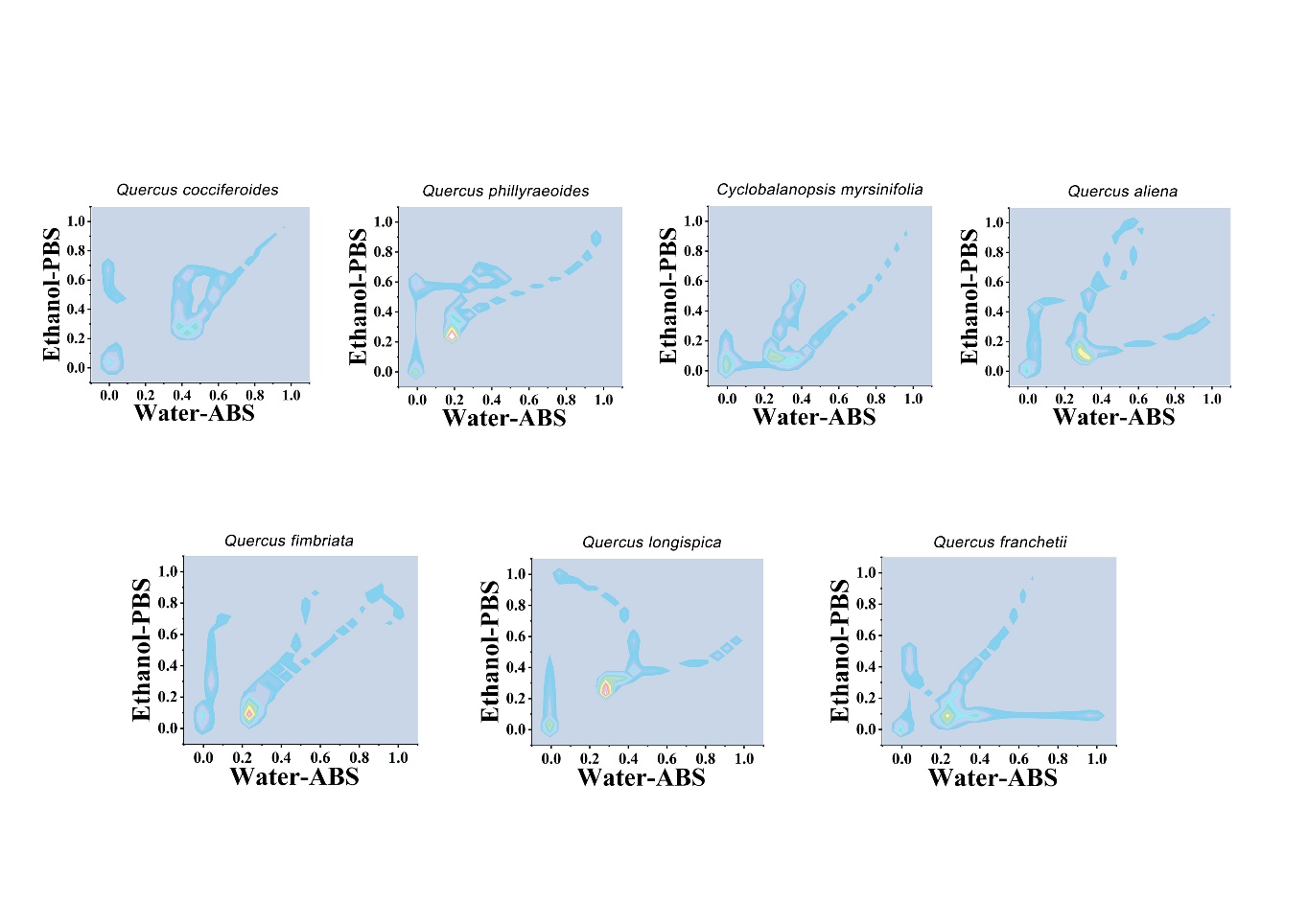


**Figure S8.** Two-dimensional density map of *Q.cocciferodies, Q. phillyraeoides, Cyclobalanopsis myrsinifolia, Q. aliena, Q. fimbriata, Q. longispica, Q. franchetli* combining the signals collected under ABS for the water extracts and under PBS for the ethanol extracts.


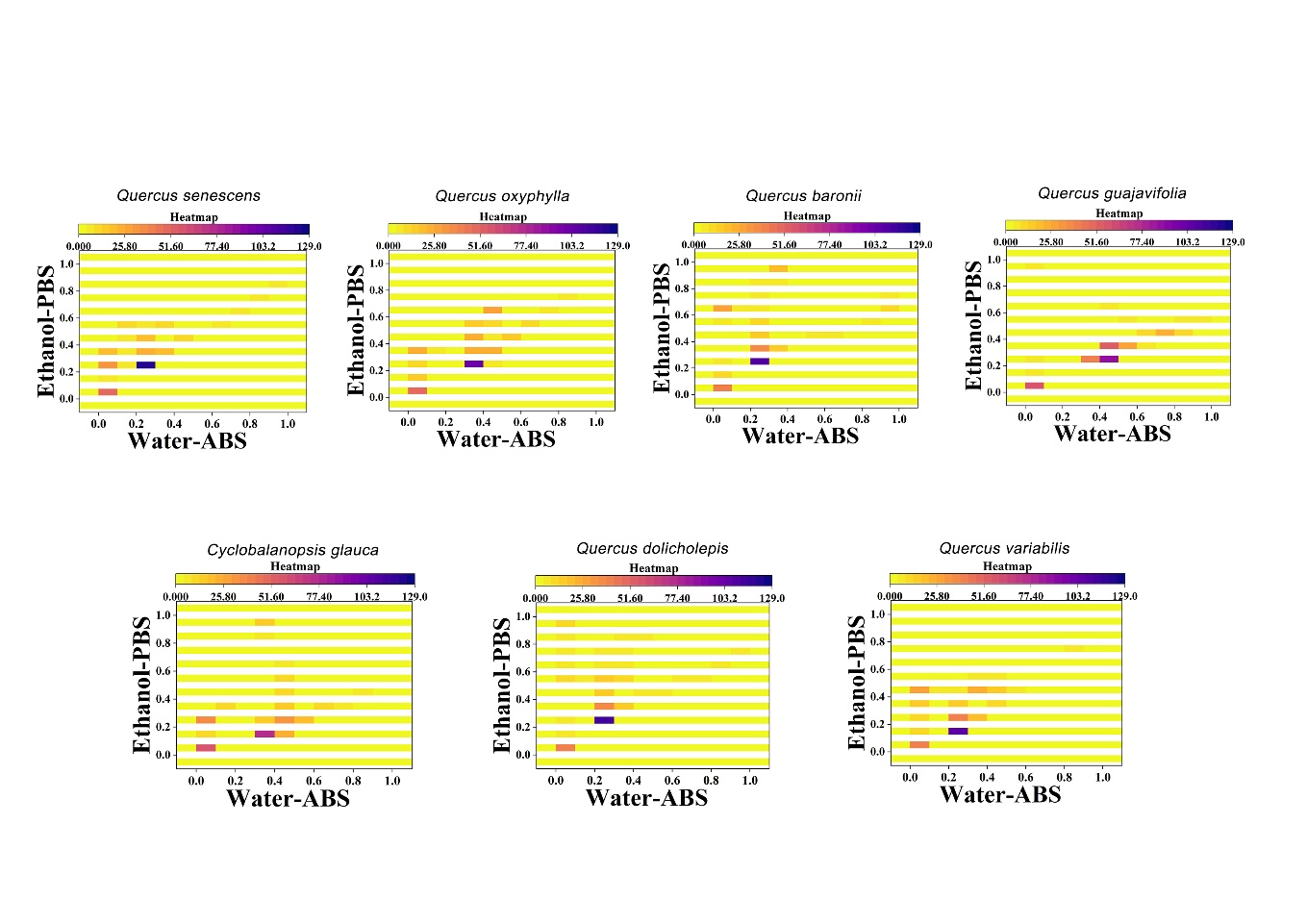


**Figure S9.** Heatmap of *Q. senescens, Q. oxyphylla, Q. baronii, Q. guajavifolia, Cyclobalanopsis glauca, Q. dolicholepis* and *Q. variabilis* combining the signals collected under ABS for the water extracts and under PBS for the ethanol extracts.


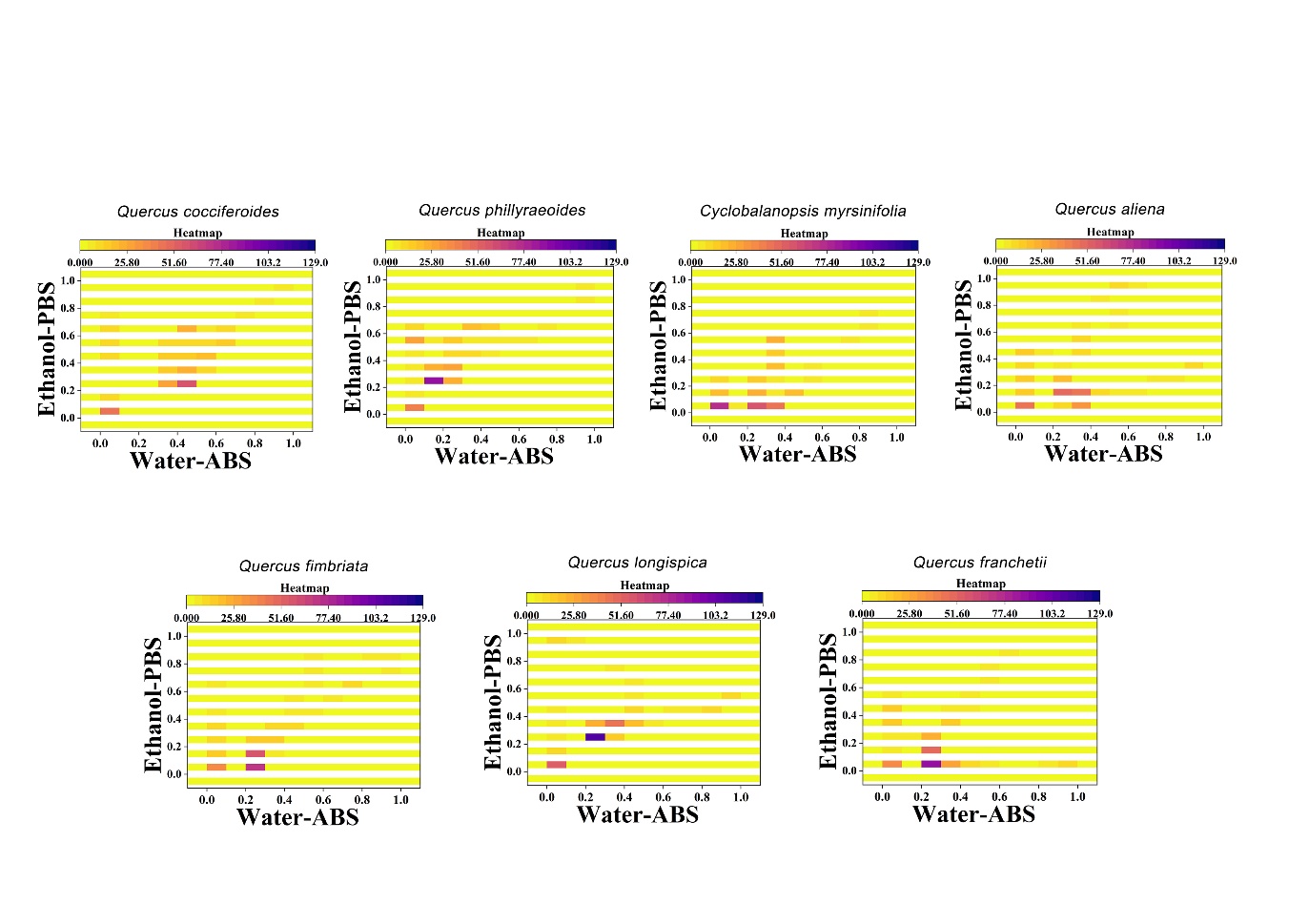


**Figure S10.** Heatmap of *Q.cocciferodies, Q. phillyraeoides, Cyclobalanopsis myrsinifolia, Q. aliena, Q. fimbriata, Q. longispica, Q. franchetli* combining the signals collected under ABS for the water extracts and under PBS for the ethanol extracts.
